# Characteristics of Microbial Communities in Crustal Fluids in a Deep-Sea Hydrothermal Field of the Suiyo Seamount

**DOI:** 10.3389/fmicb.2013.00085

**Published:** 2013-04-17

**Authors:** Shingo Kato, Michiyuki Nakawake, Junko Kita, Toshiro Yamanaka, Motoo Utsumi, Kei Okamura, Jun-ichiro Ishibashi, Moriya Ohkuma, Akihiko Yamagishi

**Affiliations:** ^1^Japan Collection of Microorganisms, RIKEN BioResource CenterWako-shi, Saitama, Japan; ^2^Department of Molecular Biology, Tokyo University of Pharmacy and Life ScienceHachioji, Tokyo, Japan; ^3^Graduate School of Natural Science and Technology, Okayama UniversityTsushima, Okayama, Japan; ^4^Graduate School of Life and Environmental Sciences, University of TsukubaTsukuba, Ibaraki, Japan; ^5^Center for Advanced Marine Core Research, Kochi UniversityNankoku, Kochi, Japan; ^6^Department of Earth and Planetary Science, Faculty of Science, Kyushu UniversityHigashi-ku, Fukuoka-shi, Fukuoka, Japan

**Keywords:** bacteria, archaea, 16S rRNA gene, crustal fluids, deep-sea hydrothermal vent, sub-seafloor biosphere, Island-Arc, Western Pacific

## Abstract

To directly access the sub-seafloor microbial communities, seafloor drilling has been done in a deep-sea hydrothermal field of the Suiyo Seamount, Izu-Bonin Arc, Western Pacific. In the present study, crustal fluids were collected from the boreholes, and the bacterial and archaeal communities in the fluids were investigated by culture-independent molecular analysis based on 16S rRNA gene sequences. Bottom seawater, sands, rocks, sulfide mound, and chimneys were also collected around the boreholes and analyzed for comparisons. Comprehensive analysis revealed the characteristics of the microbial community composition in the crustal fluids. Phylotypes closely related to cultured species, e.g., *Alteromonas, Halomonas, Marinobacter*, were relatively abundant in some crustal fluid samples, whereas the phylotypes related to *Pelagibacter* and the SUP05-group were relatively abundant in the seawater samples. Phylotypes related to other uncultured environmental clones in *Alphaproteobacteria* and *Gammaproteobacteria* were relatively abundant in the sand, rock, sulfide mound, and chimney samples. Furthermore, comparative analysis with previous studies of the Suiyo Seamount crustal fluids indicates the change in the microbial community composition for 3 years. Our results provide novel insights into the characteristics of the microbial communities in crustal fluids beneath a deep-sea hydrothermal field.

## Introduction

Microbes are expected to be widely distributed within oceanic crusts. However, little is known about the sub-seafloor biosphere because of the technical difficulties in directly sampling their habitats: what kinds of microbes are there, what function do they have, to what extent are they distributed, how abundant and how productive are they? The presence of microbial communities in aged (thousands-years-old) crustal fluids directly collected from the deep borehole have been shown by *in situ* colonization experiments and direct culture-independent molecular analysis (Cowen et al., 2003; Orcutt et al., 2011a). Considering the vast extent of the seafloor on Earth (∼70% of the total surface area), the microbes within the crusts potentially play an important role in global geochemical cycling between crusts and oceans (Schrenk et al., 2009; Orcutt et al., 2011b).

Microbial communities in crustal fluids, which were directly collected from shallow sub-seafloor environments (∼10 m depth below the seafloor) by drilling or pipe-insertion approach, have been investigated for three areas with different tectonic backgrounds, i.e., ridge flank, back-arc spreading center, and island-arc. Results from the Baby Bare seamount in the flank of the Juan de Fuca Ridge (JdFR) suggest that diverse bacteria and archaea are present in the crustal fluids (∼20°C) collected by pipe-insertion, and the microbial communities have been interpreted to be the mixture of those in the fluids, overlying seawater and sediments (Huber et al., 2006). In the Southern Mariana Trough (SMT) of a back-arc spreading center, microbial communities in the crustal fluids were different from those of the other habitats, as shown by comparative analysis of crustal fluids (6–40°C) collected from boreholes drilled by the benthic multi-coring system (BMS), bottom seawater, vent fluids, microbial mats, and sulfide chimneys (Kato et al., 2009b,c, 2010). The distinct difference found in different habitats can be explained by energy availability of each inorganic redox reaction for chemolithoautotrophs thriving there (Kato et al., 2012a). In the Suiyo Seamount of the Izu-Bonin Arc, several researchers have collected crustal fluids from BMS boreholes and characterized microbial communities in the fluids (4–112°C) by culture-independent molecular analysis (Higashi et al., 2004; Nakagawa et al., 2004; Hara et al., 2005; Kato et al., 2009a). These results have implied that the phylotypes in the crustal fluids were distinct from those in the surrounding seawater. However, uniqueness of the microbial communities for the crustal fluids has not been evaluated because no data of surrounding sediments, rocks or chimneys in the Suiyo Seamount hydrothermal field are available.

The Suiyo Seamount is an active deep-sea volcano, which lies on the Izu-Bonin Arc, Western Pacific. The seafloor in the caldera of the Suiyo Seamount is covered with sandy pumice and hydrothermal deposits. The temperature within the pumices at 10–40 cm depth ranged from 10 to 40°C in non-hydrothermal areas, while those within ∼10 m of active vents ranged from 5 to 80°C (Kinoshita et al., 2006). Heat flow measurement have indicated that local heat flow and fluid circulation occurs around active vents (Kinoshita et al., 2006). Seven boreholes (called APSK01 to APSK07) were drilled on the seafloor using BMS in June 2001, and three boreholes (APSK08 to APSK10) were added in July 2002. The analysis of core samples obtained from the boreholes have indicated that there is an impermeable layer, which consists of clay and anhydrite, at 1–3 m depth below the seafloor, and that over 300°C hydrothermal fluids are trapped below the layer (Urabe et al., 2002; Marumo et al., 2008).

**Figure 1:**
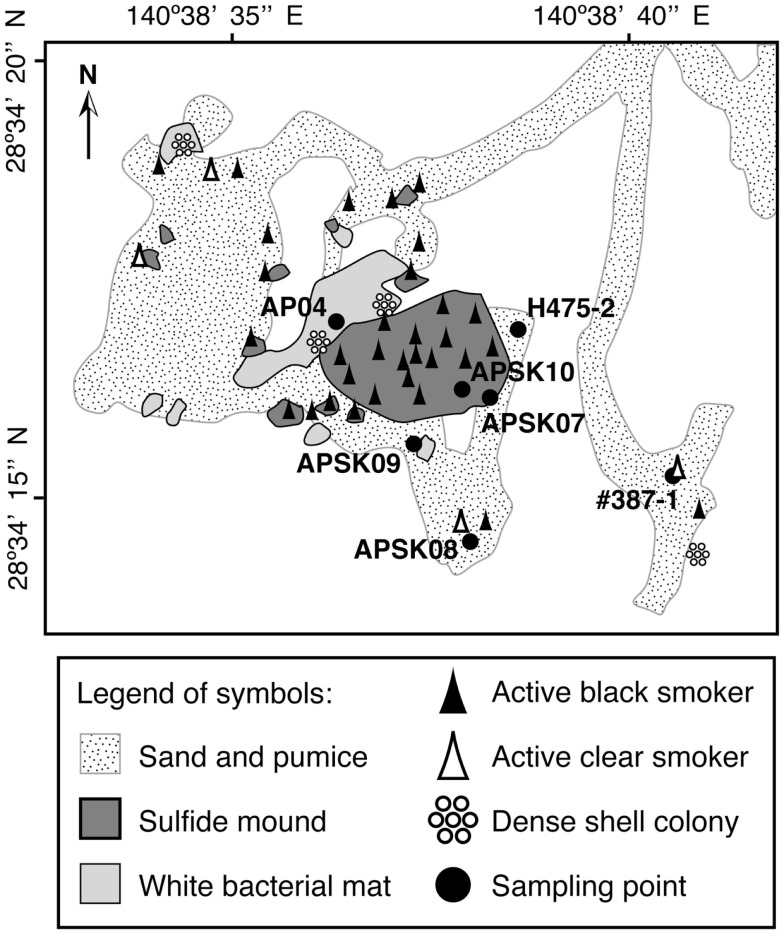
**Schematic map of the caldera bottom of the Suiyo Seamount (modified from Kato et al., 2009a)**. Legend of symbols is indicated in the bottom box.

Previously researchers have revisited the Suiyo Seamount hydrothermal field in July, August and September 2001, August and September 2002, and December 2003. The temperatures of fluids discharged from the boreholes ranged from 9 to 308°C when the boreholes were drilled, and fluctured over time (Higashi et al., 2004; Nakagawa et al., 2004; Hara et al., 2005; Kinoshita et al., 2006; Marumo et al., 2008; Kato et al., 2009a). The microbial communities in the crustal fluids collected from these boreholes in 2001 and 2002 have been previously reported (Higashi et al., 2004; Nakagawa et al., 2004; Hara et al., 2005; Kato et al., 2009a). We hypothesize that the microbial communities in crustal fluids change temporally by physicochemical fluctuation of the fluids. In 2005, we revisited the seamount and collected crustal fluids from the boreholes APSK07, APSK08, APSK09, and APSK10, in addition to the overlying bottom seawater, sandy sediments, rocks, and sulfide deposits. The locations of the sampling points are shown in Figure 1. Comprehensive analysis using these samples enables us to evaluate the characteristics of the microbial communities of the crustal fluids in this field. Furthermore, comparative analysis of the previous and present data of the Suiyo Seamount will provide information for a better understanding of temporal change of the microbial communities within the crustal aquifers caused by physicochemical fluctuation.

## Materials and Methods

### Sample collection

Fluid samples from boreholes, a natural vent and overlying seawater, and solid samples of sands, sulfide deposits, and rocks were collected in the deep-sea hydrothermal field of the Suiyo Seamount during the NT05-16 cruise (22 September to 7 October 2005) of R/V *Natsushima* (JAMSTEC, Japan) with the remotely operated vehicle *Hyper-Dolphin* (JAMSTEC, Japan). The sample IDs and other information of the collected samples are shown in Table 1. We collected the fluids from boreholes APSK07 (1,383 m water depth), APSK08 (1,386 m), APSK09 (1,387 m), APSK10 (1,380 m), and the vent AP04 (1,376 m) using fluid samplers as described previously (Kato et al., 2009c). To avoid cross-contamination from seawater, the sampling nozzle was inserted deeply into the boreholes, and the fluids were collected carefully and slowly (100 mL/min) using a peristal pump. Detailed information about APSK07, APSK09, and APSK10 has been described previously (Kato et al., 2009a). Titanium pipes were inserted into the boreholes APSK08, APSK09, and APSK10, except APSK07. To trap particles in the collected liquid samples, 1 l of the bottom seawater, and 0.3 l of the borehole and vent fluids were filtered through 0.2-μm-pore-size polycarbonate membranes (Advantec, Tokyo, Japan) using a vacuum pump on board. The filters were stored at −80°C for DNA analysis. For microscopy, aliquots of the liquid samples were fixed, filtered and stored at −20°C as described previously (Kato et al., 2009c). The rock and sulfide deposits collected by a manipulator on the vehicle were crushed into pieces using an autoclaved hammer and chisel in a clean box on board. The sands were collected using a sediment sampler that can be capped immediately after a sampling like a Niskin bottle. The solid samples were stored in DNA/RNA-free plastic tubes at −80°C until DNA extraction.

**Table 1:** **List of the samples used in this study and their characteristics**.

Sample type	Sample ID	Sampling site	Sampling date/Dive#	Geochemical characteristics^a^						Cell density (cells/ml)^c^	Bac/Arc^d^
				Temp. (°C)	pH	Si (uM)	H_2_S (mM)	Mg (mM)	Seawater%^b^	
Borehole fluid	Sm7cp	APSK07	2005.10.1/HD476	115–165^g^	4.72	8291	2.58	19.6	36.1	3.56 (0.16) × 10^5^	99.9
	Sm8cp	APSK08	2005.9.28/HD473	4.5	7.74	132	n.d.^e^	49.6	99.6	1.22 (0.04) × 10^5^	99.2
	Sm9cp	APSK09	2005.10.1/HD476	4.7	7.63	153	n.d.	50.3	98.2	1.19 (0.05) × 10^5^	>99.9
	Sm10cp	APSK10	2005.9.28/HD474	4.8	7.60	140	n.d.	50.0	98.5	0.78 (0.04) × 10^5^	99.9
Ambient seawater	Sm7sw	APSK07	2005.10.1/HD476	4.2	7.62	132	n.d.	54.3	–^f^	0.52 (0.04) × 10^5^	99.3
	Sm8sw	APSK08	2005.9.28/HD473	4.2	7.72	140	n.d.	49.8	–	1.05 (0.07) × 10^5^	99.6
	Sm10sw	APSK10	2005.9.28/HD474	4.7	7.56	156	n.d.	50.8	–	0.95 (0.04) × 10^5^	99.4
	Sm4sw	AP04	2005.9.29/HD475	4.2	7.67	133	n.d.	50.2	–	0.83 (0.07) × 10^5^	99.6
Natural vent fluid	Sm4hw	AP04	2005.9.29/HD475	6.3–27^g^	7.08	659	0.06	45.7	91.0	2.86 (0.13) × 10^5^	>99.9
Sulfide mound	Sm4sm	AP04	2005.9.29/HD475	(6.3–27)^h^	–	–	–	–	–	n.d.	98.5
Rock	Sm4rk	near A04	2005.10.1/HD476	–	–	–	–	–	–	n.d.	>99.9
Sand	Smhsd	Marker H475-2	2005.9.29/HD475	–	–	–	–	–	–	n.d.	>99.9
Active chimney	Smmcs	Marker #387-1	2005.10.1/HD476	(22–49)^h^	–	–	–	–	–	n.d.	91.4

*^a^Geochemical characteristics were determined as previously described (Nakagawa et al., 2004). ^b^Calculation based on the Mg concentration of the fluid and ambient seawater samples. ^c^Cell numbers in fluids were counted by microscopy. Numbers in parentheses indicate standard deviations of the means. ^d^Determined by Q-PCR. ^e^No data. ^f^Unapplicable. ^g^The temperature of the fluids fluctuated during the sampling. ^h^The temperature of the venting fluid*.

### Geochemical analysis

The geochemical characteristics were determined as previously described (Nakagawa et al., 2004). In brief, the temperature of fluid was determined *in situ* using a thermometer equipped with the vehicle. The pH was determined onboard using a pH electrode. The pH meter was standardized using NBS standards (pH = 6.86 and 4.01). The electrode was soaked in surface seawater for conditioning, for more than an hour till the sample measurements. The H_2_S and Si concentrations were determined onboard by colorimetry. The Mg concentration was determined using inductively coupled plasma atomic emission spectroscopy in an onshore laboratory.

### Direct cell counting

Cells on the filters were stained with SYBR Green I for 5 min. Microscopic images were collected using a fluorescence microscope BX60 (Olympus, Tokyo, Japan). To estimate the cell density, cells in at least 40 microscopic images were counted for each sample. The filtration volume of each sample was adjusted to at least 10 cells per view.

### PCR-based phylogenetic analysis

PCR-based analysis for the liquid and solid samples was performed as previously described (Kato et al., 2009b,c) with minor modifications. In brief, genomic DNA was extracted from the samples by FastDNA SPIN Kit for Soil (Qbiogene, Carlsbad, CA, USA). Partial 16S rRNA gene sequences of the whole prokaryote and *Archaea* were amplified by PCR with a prokaryote-universal primer set, Uni515F and Uni1406R (Kato et al., 2009b), and with an *Archaea*-specific primer set, Arc21F and Arc958R (DeLong, 1992). PCR amplification of *cbbM* genes using the primer set, cbbM343F and cbbM1126R, was performed as previously described (Kato et al., 2012b). The PCR products were cloned, and the nucleotide sequences of randomly selected clones were determined. The archaeal clones in the libraries from the PCR with the prokaryote-universal primer set were excluded from the analyses. The 16S rRNA gene sequences were aligned using Infernal build in Ribosomal Database Project (Cole et al., 2009). Clones having 97% sequence similarity or higher were sorted using mothur (Schloss et al., 2009) and treated as the same phylotype. Maximum likelihood (ML) trees were constructed using PHYML (Guindon and Gascuel, 2003). The taxonomic distribution of the clones was shown using VITCOMIC (Mori et al., 2010). Rarefaction analysis, Shannon diversity index and Chao1 richness estimators for each clone library, and the numbers of shared phylotypes among clone libraries were calculated using mothur. The principal coordinates analysis (PCoA) were performed using Fast UniFrac (Hamady et al., 2009).

### Quantitative PCR

Bacterial and archaeal rRNA gene copy numbers in the DNA extracts were determined by quantitative PCR (Q-PCR) as previously described (Kato et al., 2009b). Regression coefficient (*r*^2^) values of the standard curve were 0.996 and 0.992 for bacterial and archaeal analyses, respectively. Relative abundance of bacterial and archaeal cells in each sample was estimated based on their average copy numbers of 16S rRNA gene per cell, 4.13 and 1.77, respectively (Lee et al., 2009). All assays were performed in triplicate.

### Accession number

The nucleotide sequences of the phylotypes reported in this paper have been deposited in the DDBJ database under the following accession numbers: AB629061–AB629489 for bacterial 16S rRNA genes and AB629490–AB629623 for archaeal 16S rRNA genes, respectively.

## Results

### Geochemical characteristics

The temperature, pH, concentrations of Si, H_2_S, and Mg, seawater fraction of fluid samples, the cell densities and the ratio of bacteria and archaea are shown in Table 1. The borehole and vent fluid samples contained a large amount of seawater ranging from 91.0 to 99.6% by volume, except APSK07 borehole fluids (Table 1), estimated from measured Mg concentration. It should be noted that the seawater fraction in Table 1 does not simply refer to the amount of cross-contamination by seawater during sampling, but contain a certain amount of not hydrothermally altered seawater as discussed below. The fluids discharging from the APSK07 borehole and the AP04 vent obviously contained hydrothermal fluids as shown by geochemical differences such as high temperature, low pH and high concentrations of Si and H_2_S. The temperatures of the fluids from the APSK08, APSK09 and APSK10 boreholes were slightly higher (0.1 to 0.3°C) than that of the surrounding seawater.

### Microbial abundance

Relative abundance of bacteria and archaea determined by Q-PCR (Table 1) indicated that bacteria dominated in all the samples (Table 1). The cell densities of the fluid samples of the boreholes (except sm10cp) and the vent were relatively higher than that of the surrounding seawater near each borehole (Table 1).

### Diversity of 16S rRNA gene sequences

**Figure 2:**
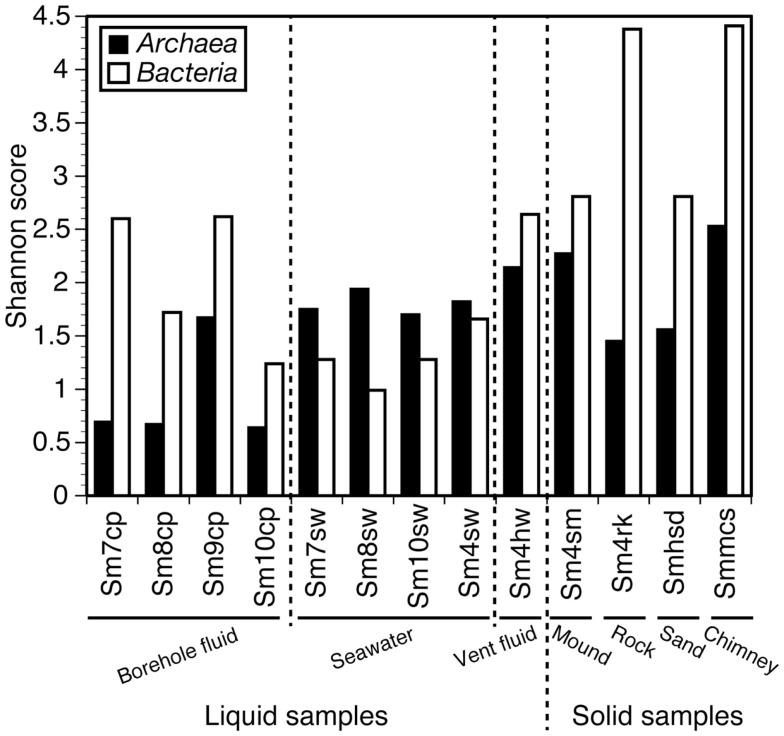
**Shannon diversity index for archaeal and bacterial 16S rRNA gene clone libraries**. Values in details are shown in Table S1 in Supplementary Material.

Shannon diversity indices of the microbial communities of the samples are shown in Figure 2 (see also Table S1 in Supplementary Material in details). When the diversity indices were compared between bacterial and archaeal communities within single samples, the archaeal communities for all the seawater samples (Sm4sw, Sm7sw, Sm8sw, and Sm10sw) showed higher diversity than the bacterial communities (1.1–2.0 times; Figure 2). The bacterial communities in the borehole fluids (Sm7cp, Sm8cp, Sm9cp, and Sm10cp), rock (Sm4rk), sand (Smhsd), and sulfide chimney (Smmcs) samples showed higher diversity (1.6–3.8 times; Figure 2) than the archaeal communities. The bacterial communities in the vent fluid (Sm4hw) and sulfide mound (Sm4sm) samples were more diverse than the archaeal communities (1.2 times; Figure 2). When the diversity indices of the bacterial communities were compared among the samples, Sm4rk and Smmcs had the highest diversity (Shannon diversity index, 4.3–4.5) and Sm8sw had the lowest diversity (<1.0) (Figure 2; Table S1 in Supplementary Material). The bacterial communities of the borehole fluid samples, except Sm10cp, showed higher diversity than those of the seawater samples (Figure 2). When the diversity indices of the archaeal communities were compared among the samples, Smmcs, Sm4sm, and Sm4hw had high diversity (Shannon diversity index, >2.1) and the seawater samples (Sm4sw, Sm7sw, Sm8sw, and Sm10sw) had moderate diversity (1.7–2.0). The borehole fluid samples, except Sm9cp, had low archaeal diversity (Shannon diversity index, <1.0) (Figure 2). Chao1 species richness estimates (Table S1 in Supplementary Material) and rarefaction curves (Figure S1 in Supplementary Material) showed the similar patterns.

### Bacterial community structures

**Figure 3:**
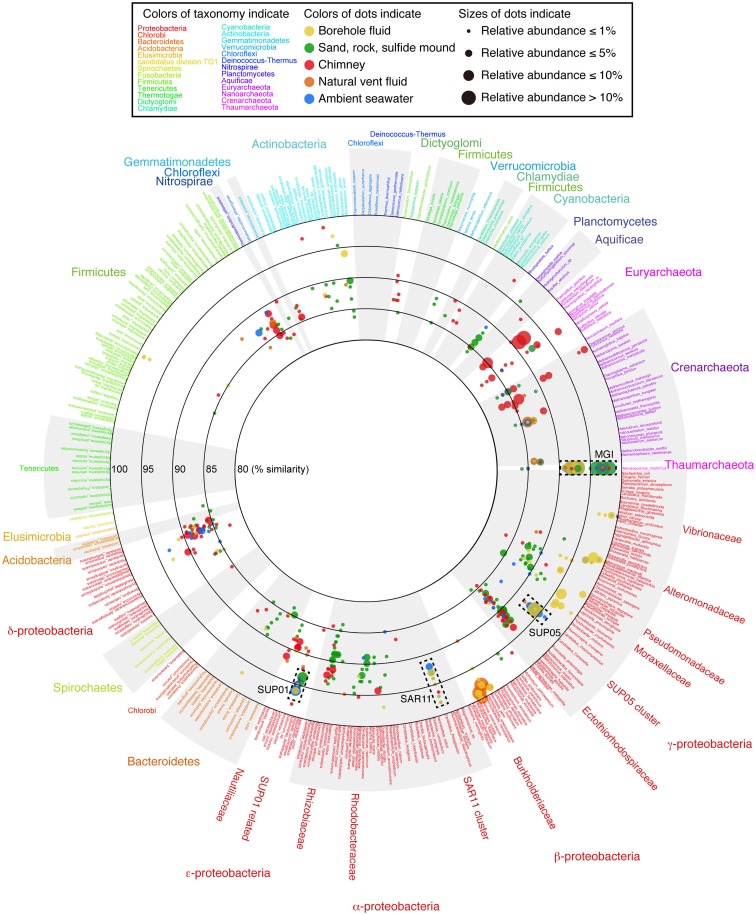
**The overview of the microbial community structures for the whole prokaryote based on 16S rRNA gene sequences visualized by VITCOMIC**. Scientific names of prokaryotic species in the reference database of VITCOMIC are shown outside of the circle. The font color for each name indicates its phylum name as shown in the box and outside of the circle. The large circles indicate boundaries of similarities (80, 85, 90, 95, and 100% similarities to the database sequence). The location of colored dots represents average similarity of full-length sequence of amplicon of each phylotype against the nearest relative species. The color of the dots indicates the sample type as shown in the box. The VITCOMIC analysis for archaeal or bacterial communities was performed separately. The total number of archaeal or bacterial sequences in the all samples is 1,011 or 1,025, respectively. The size of the dots indicates relative abundance of the phylotypes in the archaeal or bacterial clone library integrated by the sample type (smallest dot < 1%, second smallest dot < 5%, third smallest dot < 10%, and the largest dot > 10%).

To grasp the overview of the common and unique phylotypes among the samples, the microbial community structures (both archaea and bacteria) are displayed on a VITCOMIC image in Figure 3 (see also Figure S2 in Supplementary Material for enlarge views of *Alpha-*, *Beta-*, *Gamma-*, and *Epsilonproteobacteria* and *Archaea*). The image of VITCOMIC displays the taxonomic composition for not only major but also minor phylotypes in each library without loss of similarity information (Mori et al., 2010). This enables us to visually compare the microbial community structures. The taxonomic affiliation of the phylotypes related to cultured species and their relative abundance as shown in the VITCOMIC image well corresponded to that resulted from the phylogenetic tree (Figures S3 and S4 in Supplementary Material).

The recovered bacterial phylotypes were affiliated to taxonomic groups (the details are shown in Table S2 in Supplementary Material). Most phylotypes recovered from the liquid samples (i.e., the borehole fluids, vent fluid, and seawater samples) and from the sand sample were affiliated to *Proteobacteria* (82.5–100% of the total clone numbers; Table S2 in Supplementary Material). In contrast, the proportion of the proteobacterial phylotypes was relatively low (56.0–62.0%) for the rock, sulfide mound and chimney samples. Other bacterial phyla (i.e., the *Acidobacteria*, *Actinobacteria*, *Aquificae*, *Bacteroidetes*, “*Caldithrix*,” *Chlamydiae*, *Chlorobi*, *Chloroflexi*, *Cyanobacteria*, *Firmicutes*, *Gemmatimonadetes*, *Lentisphaerae*, *Nitrospirae*, *Planctomycetes*, “*Poribacteria*,” *Spirochetes*, *Thermotogae*, and *Verrucomicrobia*) and uncultured clone groups (GN02, GN06, JS1, KSB1, Marine group A, OD1, OP1, OP3, TM6, TM7, WS3) were also detected (Table S2 and Figure S3 in Supplementary Material).

**Figure 4:**
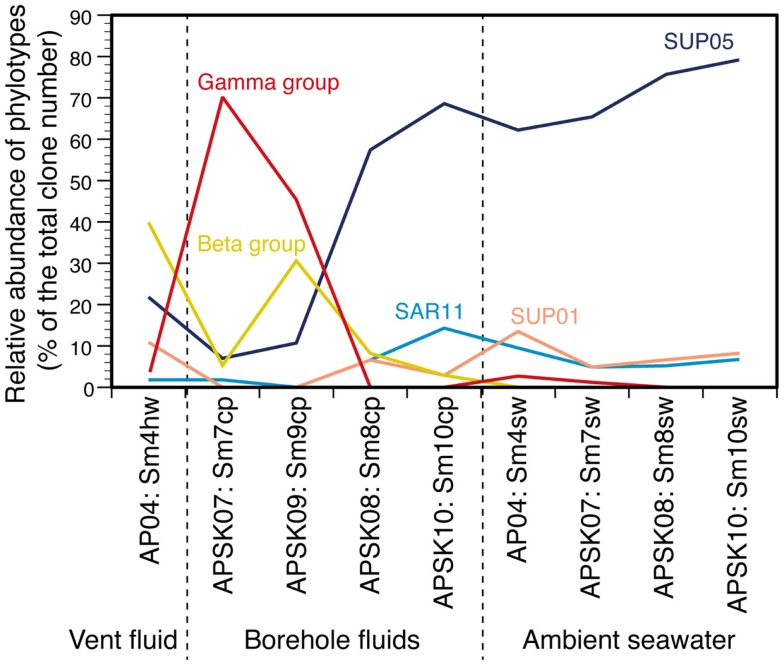
**Relative abundances of each phylotype or taxonomic group in the liquid samples**. Gamma group includes *Acinetobacter*, *Alteromonas*, *Colwellia*, *Halomonas*, *Marinobacter*, *Pseudomonas*, *Psychrobacter*, and *Vibrio*. Beta group includes *Burkholderia*, *Nitrosospira*, *Oxalobacteraceae*, *Pelomonas*, and *Ralstonia*. Values in details are shown in Table S2 in Supplementary Material.

The relative abundance of the phylotypes in several taxonomic groups is summarized in Figure 4. Many bacterial phylotypes (57.4–78.7% of the total clone numbers) recovered from Sm8cp, Sm10cp, Sm4hw, and all the seawater samples were affiliated to SUP05-related group in the *Gammaproteobacteria* (see also Table S2 and Figure S3E in Supplementary Material), which have been reported as the most abundant members in the hydrothermal plume in the Suiyo Seamount (Sunamura et al., 2004). This group has been found at other hydrothermal vent fields (Kato et al., 2009c) and oxygen minimum zone in oceans (Walsh et al., 2009), and may contain chemolithoautotrophic sulfur-oxidizing bacteria suggested by whole-genome analysis (Walsh et al., 2009). These samples also contained phylotypes related to SAR11 cluster that is a common bacterial member in oceans (Morris et al., 2002) and those related to SUP01 in the *Thiovulgaceae* that is the second abundant member in the hydrothermal plume in the Suiyo Seamount (Sunamura et al., 2004) (Figures S3A,D in Supplementary Material).

In contrast, phylotypes recovered from the other two borehole fluid samples, Sm7cp and Sm9cp, were phylogenetically close to cultured species (e.g., *Acinetobacter*, *Alteromonas*, *Halomonas*, *Marinobacter*, *Pseudomonas*, and *Vibrio* in the *Gammaproteobacteria*, and *Ralstonia* in the *Betaproteobacteria*) (Figure 3; figures S3B,E in Supplementary Material), which account for >10% of the total clone number of each library (Table S2 in Supplementary Material). The phylotypes related to *Ralstonia* and also the *Oxalobacteraceae* in the *Betaproteobacteria* were relatively abundant in the clone library from Sm4hw (21.8% of the total clone number). The SUP24-24 clone recovered from the Suiyo seamount hydrothermal plume (Sunamura et al., 2004) belongs to the *Oxalobacteraceae* (Figure S3B in Supplementary Material).

Phylotypes recovered from the solid samples, i.e., Sm4sm, Sm4rk, Smhsd, and Smmcs, had low similarity to cultured species (<95%, a general genus-level definition; Figure 3). In particular, unclassified gammaproteobacterial phylotypes were recovered from all solid samples (Table S2 in Supplementary Material; Figure 3; see also Figure S2A in Supplementary Material). These phylotypes were related to environmental clones recovered from seafloor habitats (sediment and rocks, and symbionts) in various areas and were also relatively close to chemolithoautotrophic members of *Thioprofundum* and the *Ectothiorhodospiraceae*, including sulfide-, arsenite-, and nitrite-oxidizers (Takai et al., 2009), in the phylogenetic tree (Figure S3E in Supplementary Material).

### Archaeal community structures

The recovered archaeal phylotypes were affiliated with taxonomic groups as shown in Table S3 in Supplementary Material. All phylotypes recovered from Sm7cp, Sm8cp, Sm10cp, and Sm4rk were affiliated with Marine Group I (MGI) (Figure 3; see also Figures S2C and S4 in Supplementary Material), which is one of the most abundant archaeal members in oceans (Fuhrman and Davis, 1997; Karner et al., 2001) and contain an ammonia-oxidizing isolate “*Nitrosopumilus maritimus*” (Könneke et al., 2005). In addition to MGI, phylotypes related to pSL12-related group, Marine Group II and III (MGII and MGIII) or Marine Benthic Group E (MBGE) in the *Euryarchaeota* were recovered from Sm9cp, Smhsd, and all the seawater samples (Table S3 and Figure S4 in Supplementary Material). The libraries from Sm4sm and Smmcs contained phylotypes related to (hyper)thermophilic members of *Archaeoglobi* and *Thermoprotei*. Furthermore, phylotypes related to “*Aciduliprofundum*,” *Thermococci*, and Marine Benthic Group D (MBGD) in *Euryarchaeota*, *Korarchaeota*, and ambiguous groups, i.e., Hot Water Crenarchaeota Group (HWCG), Marine Benthic Group B (MBGB), Marine Hydrothermal Vent Group I (MHVG-I), and Terrestrial Hot Spring Crenarchaeota (THSC), which did not clearly clustered in above archaeal phyla in the phylogenetic tree (Figure S4B in Supplementary Material), were recovered only from Smmcs (Table S3 in Supplementary Material).

### Comparison of bacterial and archaeal community structures

**Figure 5:**
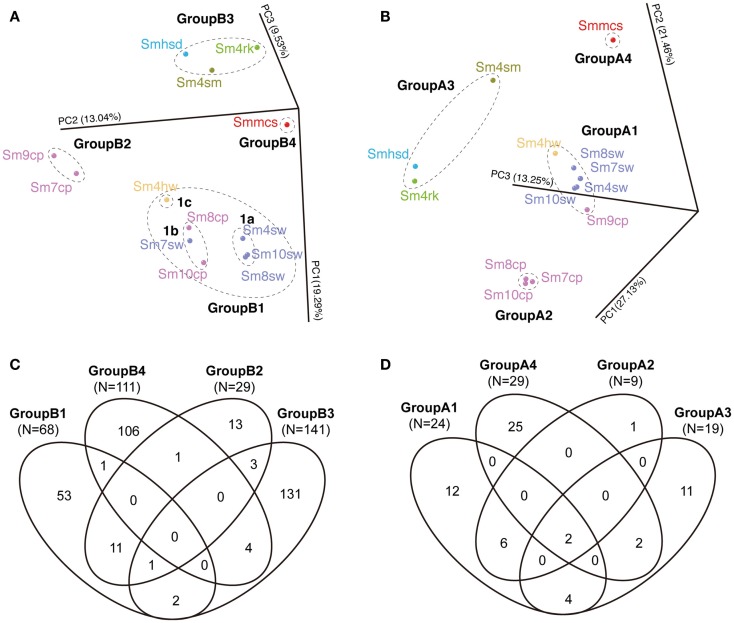
**Comparison of bacterial and archaeal community structures of the Suiyo Seamount**. The results of **(A,B)** PCoA and **(C,D)** Venn diagrams indicating the shared phylotype numbers among the groups defined in the PCoA are shown. The results of the comparative analyses for **(A,C)** bacterial and **(B,D)** archaeal communities are shown. **(A,B)** The percentages in the axis labels represent the percentages of variation explained by the principal coordinates. **(A,B)** The color of the sample name indicates each sample type: purple, borehole fluid; orange, natural vent fluid; ultramarine, seawater; green, rock; sky blue, sand; olive, sulfide mound; red, active chimney.

The communities were divided into four groups each in archaeal and bacterial communities in PCoA (Figures 5A,B). The bacterial four groups are: GroupB1 consisted of all the seawater samples (Sm4sw to Sm10sw), vent fluid sample (Sm4hw) and two borehole samples (Sm8cp and Sm10cp); GroupB2 consisted of the other borehole samples (Sm7cp and Sm9cp); GroupB3 consisted of the sand (Smhsd), rock (Sm4rk), and sulfide mound (Sm4sm) samples; and GroupB4 consisted of the chimney sample (Smmcs). The archaeal four groups are: GroupA1 consisted of all the seawater samples, Sm4hw and Sm9cp; GroupA2 consisted of Sm7cp, Sm8cp, and Sm10cp; and GroupA3 and GroupA4 consisted of the same samples as in the case of GroupB3 and GroupB4 for bacterial communities.

The shared phylotype numbers among the groups are shown in Venn diagrams (Figures 5C,D). Over half of the bacterial phylotypes in GroupB3 (92.9% of the total phylotype number) and GroupB4 (95.5%) and archaeal phylotypes in GroupA3 (57.9%) and GroupA4 (86.2%) were unique. These groups were all derived from the communities in the solid samples. In addition, many bacterial phylotypes (70.4–95.6%) were unique among each solid sample (Figure S5A in Supplementary Material). In contrast, relatively small numbers of the phylotypes in GroupB2 (37.9% of the total phylotype number) were shared with GroupB1 (16.2%) and those in GroupA2 (66.7%) were shared with GroupA1 (25.0%); these groups were derived from the communities in the liquid samples (Figure 5). Furthermore, GroupB1 was divided into three subgroups, GroupB1a, b and c (Figure 5A). The shared phylotype numbers among GroupB1a, b, c, and GroupB2 are shown in Figure S5B in Supplementary Material. Although these (sub)groups were similar each other in the PCoA result (Figure 5A), unique phylotypes constituted a half or more of the total phylotype numbers (Figure S5B in Supplementary Material).

### Change in microbial communities in crustal fluids for 3 years

The microbial community structures in the crustal fluids collected from the boreholes in the Suiyo Seamount in 2001 and 2002 have already been reported (Higashi et al., 2004; Nakagawa et al., 2004; Kato et al., 2009a). Comparative analysis between the data in 2001–2002 and 2005 samples provide a unique opportunity to address the change of microbial communities in crustal fluids. The difference in the community compositions in the crustal fluids of the Suiyo Seamount is shown in Figure S6A in Supplementary Material by VITCOMIC. Remarkably, the phylotype related to *Thiomicrospira* was detected in the borehole fluids in 2001 and 2002 (Higashi et al., 2004; Nakagawa et al., 2004; Kato et al., 2009a); however no or few phylotypes related to these members were detected in 2005. In contrast, in 2005, the phylotypes related to gammaproteobacterial genera such as *Alteromonas*, *Pseudomonas*, and *Marinobacter* accounted for relatively high percentages in the clone libraries from the borehole fluids (Sm7cp and Sm9cp; Table S2 in Supplementary Material).

**Figure 6:**
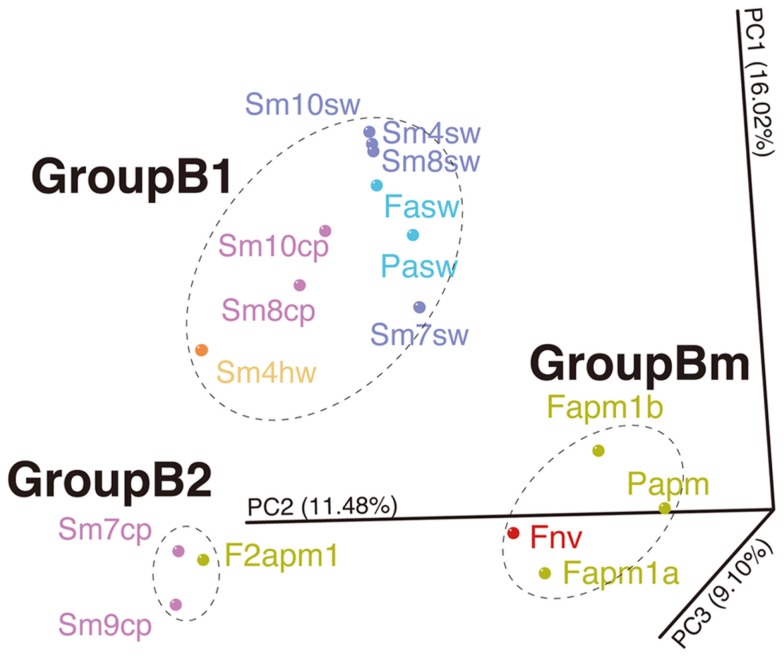
**Comparison of bacterial communities between the Suiyo Seamount and Southern Mariana Trough**. The results of PCoA are shown. Data from the previous reports of the Southern Mariana Trough (Kato et al., 2009c) are included in this analysis: Fapm1a, Fapm1b, Papm, F2apm1, Fnv, Fasw, and Pasw. The percentages in the axis labels represent the percentages of variation explained by the principal coordinates.

Likewise, such change in microbial communities has been observed in the borehole fluids in the SMT between 2004 and 2005 (Kato et al., 2009c) (Figure S6B in Supplementary Material). The analytical processes, such as the seafloor drilling, collecting fluids, and DNA analysis, has been performed by almost the same methods, so that we could compare the results of the Suiyo Seamount with those of the SMT in minimized methodological bias. The results of PCoA including the data of the Suiyo Seamount and SMT indicated that the community of the SMT borehole fluid collected in 2005 (as F2apm1 in Figure 6) was clustered within the GroupB2 (Figure 6). These communities in GroupB2 include phylotypes related to cultured *Beta*- and *Gammaproteobacteria* (Figure S6 in Supplementary Material). This grouping suggested that the characteristics of bacterial community structures in crustal fluids might converge for at least 1 year after drilling despite the difference in geographical (spatial distance) and geological settings (island-arc vs. back-arc spreading center). The communities of the SMT vent and borehole fluids collected in 2004 were clustered within GroupBm that is defined in Figure 6, which represents phylotypes related to *Thiomicrospira* and *Mariprofundus* (sulfide- and iron-oxidizing bacteria, respectively) (Kato et al., 2009c). The Suiyo Seamount borehole fluid collected in 2002 may be clustered in the GroupBm because of the representative phylotypes related to *Thiomicrospira* (Figure S6A in Supplementary Material; Kato et al., 2009a); however, we could not include these 2002 data into PCoA because only partial sequences were determined.

## Discussion

Although microbes within the crustal aquifers potentially play a role in global geochemical cycling, knowledge of these sub-seafloor microbes, i.e., their abundance, diversity, function, activity, and productivity, is still extremely limited because of the technical difficulties in directly access their habitats. Seafloor drilling provides a unique opportunity to approach microbes within crustal aquifers. Our comprehensive analysis of the 16S rRNA gene sequences (especially for bacteria) picks up the difference and commonality of the characteristics of microbial communities in each habitat-type (i.e., borehole fluid, vent fluid, ambient seawater, sand, rock, sulfide mound, and chimney) in the Suiyo Seamount, a model of the island-arc hydrothermal fields. Furthermore, comparative analysis including the previous results of the Suiyo Seamount in 2002 (Kato et al., 2009a) and of the SMT in 2004 and 2005 (Kato et al., 2009c) allows us to discuss the difference in microbial communities in the crustal fluids.

### Characteristics of microbial communities in crustal fluids

The crustal fluid communities are likely to be distinguished from the communities in the seawater and solid habitats, such as rocks. Our comparative analysis shed light on the characteristics of the crustal fluid communities in 2005. The cell densities in the borehole fluids are comparable to or slightly higher than those in ambient seawater (Table 1). The cell densities in the overlying seawater and borehole fluids is comparable to the previous reports of the JdFR flank (0.23–0.85 and 0.38–1.30 × 10^5^ cells/mL, respectively) (Cowen et al., 2003; Huber et al., 2006) and to those of the SMT (0.17–0.18 and 0.66–1.03 × 10^5^ cells/mL, respectively) (Kato et al., 2009c).

A characteristic of the crustal fluid communities of Sm7cp and Sm9cp samples is the relatively high abundance of phylotypes closely related to cultured species in *Gammaproteobacteria* (e.g., *Alteromonas*, *Halomonas*, *Marinobacter*, *Pseudomonas*, and *Vibrio*). This characteristic is one of the reasons for the separation between the crustal fluid communities (Sm7cp and Sm9cp) and the others in PCoA (Figure 5A). *Halomonas* and *Marinobacter* are widely distributed in low temperature hydrothermal vent fields (Kaye et al., 2011). These facts support that the gammaproteobacterial members in Sm7cp and Sm9cp may be present there and adapt to the low temperature crustal fluid environments. The temperature of APSK07 borehole fluids dramatically increased for 3 years (55–165°C). Despite the high temperature of the fluid sample Sm7cp, no phylotypes related to known thermophilic members were observed in the Sm7cp library. We interpret that the detected phylotypes in the Sm7cp may be transported from low temperature sub-seafloor environments. Organic carbon for the above-mentioned heterotrophic bacteria, e.g., *Alteromonas* and *Halomonas*, might be produced by chemolithoautotrophs (e.g., *Thiomicrospira*) during the first year after the drilling (Kato et al., 2009a) and/or by those (e.g., *Archaeoglobus*) in hot deeper sub-seafloor environments (Higashi et al., 2004; Hara et al., 2005). It should be noted that we have shown the presence of a variety of putative chemolithoautotrophs in the solid samples (i.e., Sm4rk, Smhsd, and Smmcs), the seawater and vent fluid samples (i.e., Sm8sw, Sm10sw, Sm4sw, and Sm4hw) (Kato et al., 2012b) used in the present study by PCR-based analysis of *cbbM* gene encoding form II of ribulose-1,5-bisphosphate carboxylase/oxygenase (RubisCO), a key enzyme in the Calvin–Benson–Basham cycle. However, *cbbM* and other genes related to carbon fixation (i.e., *cbbL* and *aclB*) have not been detected in the borehole fluid samples.

Archaeal communities seem to be different between the borehole fluid samples (except Sm9cp) and the seawater samples (Figure 5B). This likely results from the absence of MGI α-cluster and MGII in these borehole fluids (Table S3 and Figure S4 in Supplementary Material), although most of the archaeal phylotypes in the borehole fluids were also found in the seawater (Figure 5D). In contrast, the archaeal communities were clearly different between the liquid samples (representing GroupA1 and 2) and the solid samples (representing GroupA3 and 4) (Figures 5B,D). This difference is likely caused by the presence/absence of MGI ε − ζ − θ cluster and of the other archaeal groups (except MGII and pSL12-related) (Table S3 in Supplementary Material). The presence/absence of these archaeal groups may reflect the temperature, pH, and availability of energy sources associated with hydrothermal activity as discussed previously (Kato et al., 2010), as well as their life-style, i.e., free-living or attached to a solid support as reported in other marine environments (Durbin and Teske, 2010; Nitahara et al., 2011).

Our results show the characteristics of the bottom seawater communities: (i) low cell density (<1.1 × 10^5^ cells/mL); (ii) low phylogenetic diversity (<2.0 of Shannon diversity index); (iii) higher diversity of archaea than bacteria (1.1–2.0 times); and (iv) high relative abundance of the phylotypes of the SUP05-related group in *Gammaproteobacteria* (60–80% of the total clone numbers), SUP01 cluster in *Epsilonproteobacteria* (5–15%), and SAR11 cluster in *Alphaproteobacteria* (5–10%). These characteristics are partially consistent with those reported for the bottom seawater of the Suiyo Seamount sampled in 2002 (Kato et al., 2009a) and those of the back-arc spreading center deep-sea hydrothermal fields of the SMT (Kato et al., 2009c), suggesting that these characteristics of the bottom seawater communities are common at deep-sea water around hydrothermal fields in island-arc and back-arc systems. Microbial communities in the habitats of the solid samples are characterized as: (i) moderate or high phylogenetic diversity (Shannon diversity index, 2.8–4.3); (ii) relatively high abundance of the phylotypes related to uncultured bacteria including *Proteobacteria* and other phyla; and (iii) distinct community structures among the habitats (i.e., sands, rocks, sulfide mounds, and chimneys). These characteristics are consistent with those of the SMT (Kato et al., 2009b, 2010). Overall, our results suggest that the characteristics of the microbial communities of overlaying bottom seawater and surrounding solid habitats are different from those of crustal fluids in island-arc and back-arc systems.

### The change in microbial community compositions in crustal fluids for 3 years

**Figure 7:**
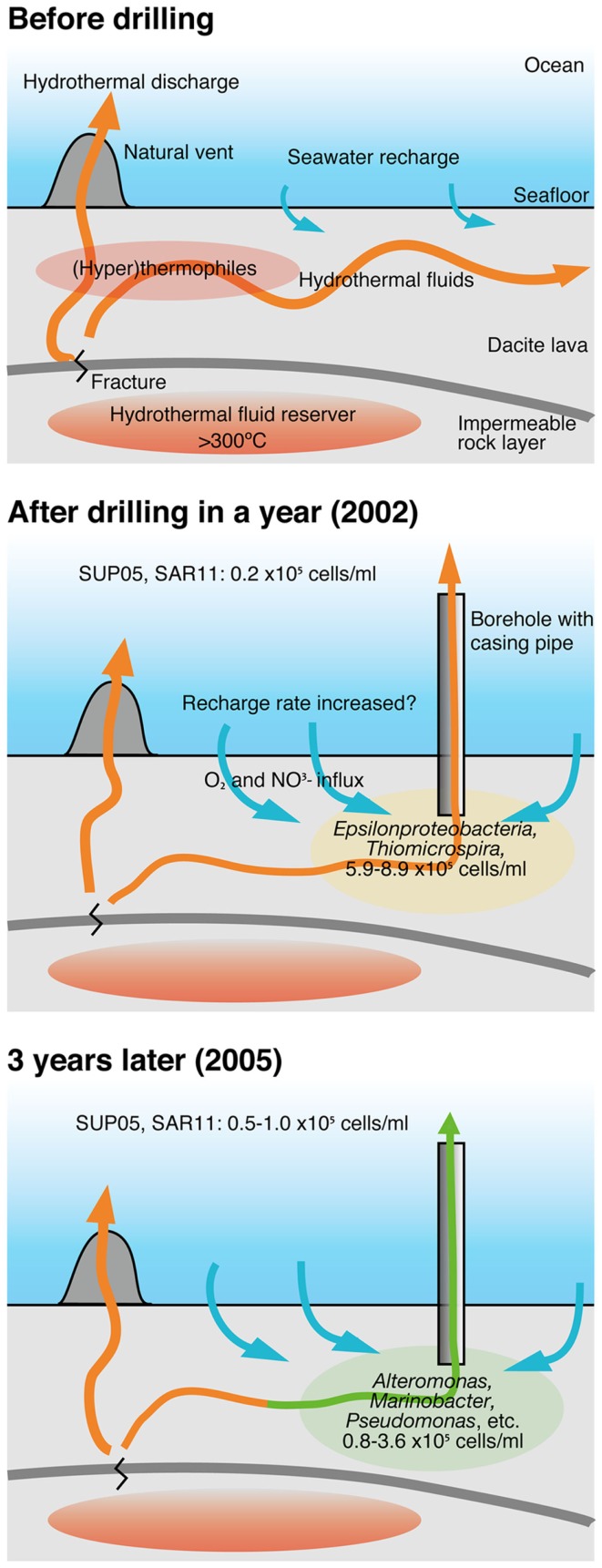
**A model of the change of microbial communities in crustal fluids**. This model is based on comprehensive results from geological, geochemical and microbiological studies of the hydrothermal field of the Suiyo Seamount. See text in details. Arrows indicate fluid flow.

The changes in the community compositions in the crustal fluids of the Suiyo Seamount for 3 years are summarized (Figure 7) based on comprehensive data regarding the geological and geochemical characterization as described previously (Marumo et al., 2008; Toki et al., 2008). Although putative sulfide-oxidizing chemolithoautotrophs related to *Thiomicrospira* were relatively abundant in the borehole fluids in 2001 and 2002 (Higashi et al., 2004; Nakagawa et al., 2004; Kato et al., 2009a), putative heterotrophs related to such as *Alteromonas*, *Pseudomonas*, and *Marinobacter* were relatively abundant in the borehole fluids in 2005 (Figure S6A in Supplementary Material). Considering the inferred physiological characteristics of the detected phylotypes, the changes in microbial communities in the crustal fluids for 3 years may be caused by the changes of geochemical characteristics of the crustal fluids. As compared with 2002 data, the increase in pH (6.6–7.6) and decrease in concentrations of H_2_S (0.74 mM to not detected) was observed for APSK09 borehole fluids. The decrease in H_2_S is consisted with the decrease in the relative abundance of the phylotypes related to *Thiomicrospira*.

It is possible that the geochemical changes are caused by the creation of new discharging points using the drilling process. The creation of a discharging point is expected to lead to more recharge of the seawater and to oxygenation of the sub-seafloor habitats. During the first year after drilling, phylotypes related to *Thiomicrospira* were present in sub-seafloor, and might utilize reduced-sulfides contained in hydrothermal fluids and oxygen and/or nitrate contained in recharged seawater (Figure 7; Kato et al., 2009a). Heat flow measurements indicate that local recharges of seawater into the seafloor occur in the Suiyo Seamount (Kinoshita et al., 2006). After the depletion of these electron donors (i.e., reduced-sulfides) by microbial consumption and/or chemical precipitation, putative heterotrophic bacteria could thrive utilizing organic carbon as described above. Our hypothesis can clearly explain the observed change in the microbial communities in the crustal fluids of the Suiyo Seamount from 2002 to 2005 and also of the SMT from 2004 to 2005 (Figure 6). The change in the microbial communities potentially occurs in global sub-seafloor environments from the opening of a new hydrothermal vent to the ceasing of hydrothermal activity. Further investigations, e.g., physiological characterization of these phylotypes by cultivation and/or metagenomic approaches, *in situ* measurement of the metabolic reaction and detailed geochemical characterization of the crustal fluids over time, are needed to assess this hypothesis.

## Conflict of Interest Statement

The authors declare that the research was conducted in the absence of any commercial or financial relationships that could be construed as a potential conflict of interest.

## Supplementary Material

The Supplementary Material for this article can be found online at http://www.frontiersin.org/Extreme_Microbiology/10.3389/fmicb.2013.00085/abstract
